# Quercetin as a possible complementary agent for early-stage COVID-19: Concluding results of a randomized clinical trial

**DOI:** 10.3389/fphar.2022.1096853

**Published:** 2023-01-13

**Authors:** Francesco Di Pierro, Amjad Khan, Somia Iqtadar, Sami Ullah Mumtaz, Muhammad Nabeel Akbar Chaudhry, Alexander Bertuccioli, Giuseppe Derosa, Pamela Maffioli, Stefano Togni, Antonella Riva, Pietro Allegrini, Martino Recchia, Nicola Zerbinati

**Affiliations:** ^1^ Scientific and Research Department, Velleja Research, Milan, Italy; ^2^ Digestive Endoscopy, Fondazione Poliambulanza, Brescia, Italy; ^3^ INEOS Oxford Institute for Antimicrobial Research, University of Oxford, Oxford, United Kingdom; ^4^ Department of Medicine, King Edward Medical University, Lahore, Pakistan; ^5^ Punjab Institute of Cardiology, Lahore, Pakistan; ^6^ Department of Biomolecular Sciences, University of Urbino, Urbino, Italy; ^7^ Department of Internal Medicine and Therapeutics, University of Pavia, Pavia, Italy; ^8^ Centre of Diabetes and Metabolic Diseases, Department of Internal Medicine and Therapeutics, University of Pavia, Pavia, Italy; ^9^ R&D Department, Indena S.p.A, Milan, Italy; ^10^ Medistat, Milan, Italy; ^11^ Department of Medicine and Surgery, University of Insubria, Varese, Italy

**Keywords:** Quercetin, COVID-19, SARS-CoV-2, Phytosome^®^, Natural Polyphenols, 3CL protease inhibition

## Abstract

**Background:** Quercetin, a natural polyphenol with demonstrated broad-spectrum antiviral, anti-inflammatory, and antioxidant properties, has been proposed as an adjuvant for early-stage coronavirus disease 2019 (COVID-19) infection.

**Objective:** To explore the possible therapeutic effect of quercetin in outpatients with early-stage mild to moderate symptoms of COVID-19.

**Methods:** This was an open-label randomized controlled clinical trial conducted at the department of medicine, King Edward Medical University, Lahore, PK. Patients were randomized to receive either standard of care (SC) plus an oral quercetin supplement (500 mg Quercetin Phytosome®, 1st week, TDS: 2nd week, BDS) (*n* = 50, quercetin group) or SC alone (*n* = 50, control group).

**Results:** After one week of treatment, patients in the quercetin group showed a speedy recovery from COVID-19 as compared to the control group, i.e., 34 patients (vs. 12 in the control group) tested negative for severe acute respiratory syndrome coronavirus 2 (SARS-CoV-2) (*p* = 0.0004), and 26 patients (vs. 12 in the control group) had their COVID-19-associated acute symptoms resolved (*p* = 0.0051). Patients in the quercetin group also showed a significant fall in the serum lactate dehydrogenase (LDH) mean values i.e., from 406.56 ± 183.92 to 257.74 ± 110.73 U/L, *p* = 0.0001. Quercetin was well-tolerated by all the 50 patients, and no side effects were reported.

**Conclusion:** Our results, suggest the possible therapeutic role of quercetin in early-stage COVID-19, including speedy clearance of SARS-CoV-2, early resolution of the acute symptoms and modulation of the host’s hyperinflammatory response.

**Clinical Trial Registration:** clinicaltrials.gov, identifier NCT04861298

## Introduction

Coronavirus disease 2019 (COVID-19), a pathology caused by the novel severe acute respiratory syndrome coronavirus 2 (SARS-CoV-2), is a respiratory viral infectious disease that has provoked an ongoing pandemic. COVID-19 has severely affected the lives of all human beings. As of 7 July 2022, the disease has affected more than 552 million people and resulted in nearly 6.34 million deaths worldwide. Although effective COVID-19 vaccines have been developed, but possible mutations in the SARS-CoV-2 put the effectiveness of the vaccination campaign at serious risk. There is currently no specific and conclusively proven treatment available for COVID-19. Though several early-stage COVID-19 antivirals including molnupiravir, sotrovimab, casirivimab/imdevimab, nirmatrelvir/ritonavir have been developed, but these drugs are highly costly and available only in a few developed countries such as the United Kingdom and United States. Reported evidence suggest that it is highly unlikely that a single “magic bullet” drug will cure COVID-19, but rather a combination therapy of antiviral and anti-inflammatory agents administered at the *early-stage* is likely to be an effective treatment for COVID-19 (Chakraborty et al., 2021).

**FIGURE 1:**
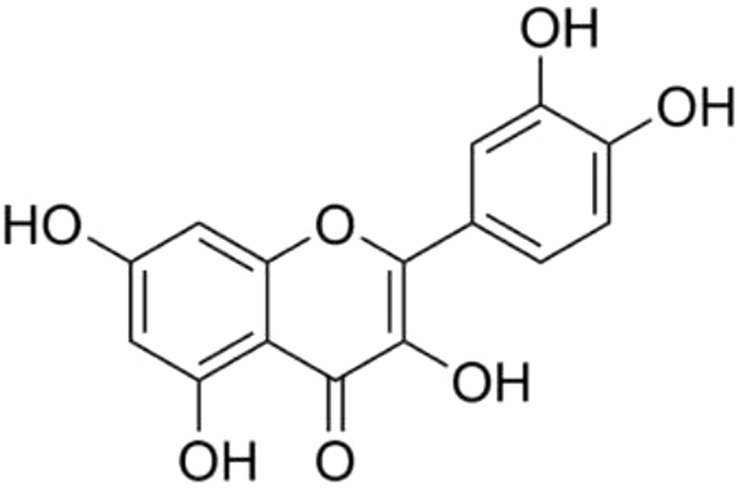
Chemical structure of quercetin.

There is currently a wide scientific interest to explore natural dietary supplements as possible adjuvant therapy for COVID-19. Amongst such agents is quercetin **(** Figure 1), an extensively studied flavonoid, that possesses diverse pharmacological activities, including antioxidant, anti-inflammatory, immunomodulatory, and anti-senescence (Colunga Biancatelli et al., 2020; Derosa et al., 2021; Lee et al., 2021; Saeedi-Boroujeni and Mahmoudian-Sani, 2021). Quercetin has shown inhibitory activity against many viruses including SARS-CoV-2 (Abian et al., 2020a; Kandeil et al., 2021; Rizzuti et al., 2021; Bahun et al., 2022), SARS-CoV-1 (Chen et al., 2006), human respiratory syncytial virus, human immunodeficiency virus (HIV), poliovirus (type 1), herpes simplex virus (type 1 and 2), Hepatitis B and C virus, and parainfluenza virus (type 3) (Colunga Biancatelli et al., 2020; Derosa et al., 2021; Di Petrillo et al., 2022). Recently, several clinical trials have revealed treatment benefits of quercetin supplementation in patients with COVID-19, including prophylaxis, speedy clearance of the SARS-CoV-2, speedy resolution of the acute symptoms and improvement in the serum inflammatory biomarker levels (Imran et al., 2022). Quercetin has a well-demonstrated safety and tolerability profile in humans and has received the FDA GRAS (Generally Recognized As Safe) status for use as a dietary supplement (US Food and Drug Administration Letter, 2010). In the middle of this ongoing pandemic, we investigated the possible treatment benefits of quercetin in mild to moderately symptomatic COVID-19 outpatients in a randomized clinical trial with an aim that quercetin is safe, cheap, and worldwide available agent, and could help in the care of community-based COVID-19 patients (Di Pierro et al., 2021a). In this study, patients who received quercetin (500 mg oral Quercetin Phytosome® BDS for 30 days) supplement as add-on to the standard of care (SC) (*n* = 76) showed a significant reduction in the rate and length of hospitalization, need of non-invasive oxygen therapy, progression to intensive care unit and mortality. The study also evaluated the excellent safety and tolerability profile of quercetin in patients with COVID-19. The promising results of this first clinical trial prompted us to investigate if quercetin supplementation could possibly affect the time to SARS-CoV-2 clearance and improvement in the COVID-19-associated acute symptoms in a second clinical trial in mild to moderately symptomatic COVID-19 outpatients. Due to the urgent requirement for *early-stage* COVID-19 medications, in May 2021, we reported the preliminary results of the first 42 patients enrolled in this study (Di Pierro et al., 2021b). The results revealed that patients treated with quercetin (500 mg Quercetin Phytosome® TDS for the 1^st^ week and BDS for the 2^nd^ week) as add-on to the SC, had a shorter time to SARS-CoV-2 clearance as shown by reverse-transcriptase polymerase chain reaction (RT-PCR) test, an early resolution of the COVID-19-associated acute symptoms and an improvement in the serum inflammatory biomarker levels as compared to the patients who received the SC alone. In the present study we report the overall results of 100 COVID-19 outpatients who completed the study, and the concluding results validates the preliminary results we reported earlier, implying the possible therapeutic role of quercetin supplementation in patients at *early-stage* of COVID-19.

## Materials and methods

This was a single-center, open-label, and randomized controlled clinical trial, conducted at the Department of Medicine, King Edward University, Lahore, Pakistan. The study compared the treatment effect of SC plus quercetin vs. SC alone in mild to moderately symptomatic, RT-PCR confirmed COVID-19 outpatients. The study was approved by the Institutional Review Board King Edward Medical University, Lahore, Pakistan (PK), *via* Ref. No. 192/RC/KEMU and conducted according to the guidelines and recommendations of Good Clinical Practice and the Declaration of Helsinki. Informed written consent was obtained from each participant before enrolling in the study. The study has been registered on clinicaltrial.gov with identifier number as NCT04861298.

**FIGURE 2:**
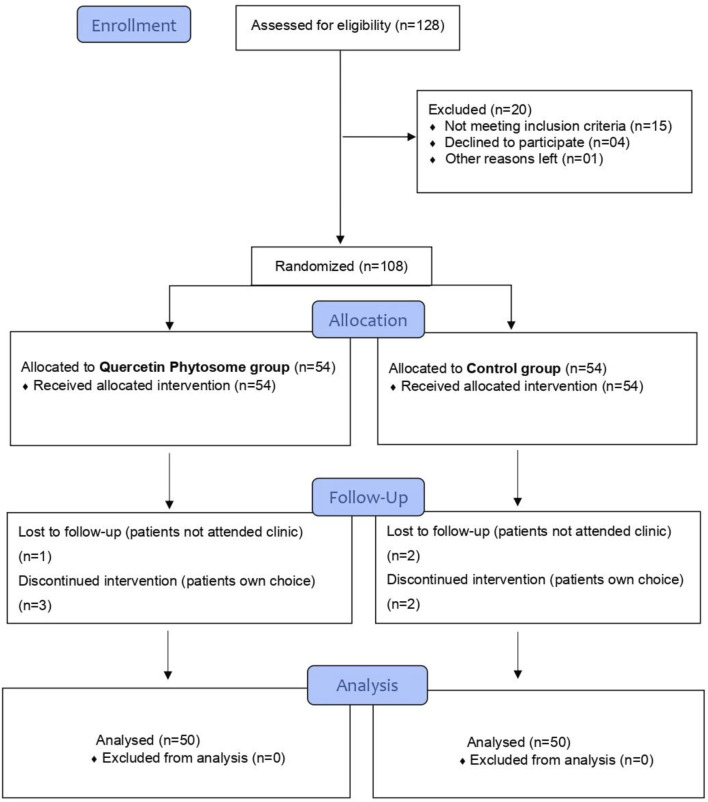
Study CONSORT flowchart.

The inclusion criteria include patients 18 years or older of either gender, tested positive for SARS-CoV-2 by nasopharyngeal and/or oropharyngeal swab RT-PCR test, with mild to moderate COVID-19 acute symptoms such as fever, dyspnea, persistent dry cough, sore throat, myalgia, weakness, oxygen saturation >93%, and not severely ill to require hospital admission. The exclusion criteria include: patients with a proven history of hypersensitivity/allergic reaction to quercetin, chronic kidney disease, severe hypotensive, moderate or severe thrombocytopenia, pregnancy, or on immune system booster medications at the time of enrolment. A total of 108 COVID-19 outpatients who attended the medical outpatients’ clinics of King Edward Medical University Lahore, PK, from December 2020 to September 2021, after fulfilling all the inclusion criteria and non-of the exclusion ones, consented, and, were enrolled in the study. Patients were randomly assigned (by Block Randomization Algorithm) in a 1:1 ratio to receive either the SC plus quercetin (quercetin group) or SC alone (control group). The randomization was carried out by a trained healthcare professional who has no role in the study. The study CONSORT flow diagram is shown in Figure 2. Patients were then clinically assessed by the outpatient’s physician for COVID-19-associated acute symptoms, and laboratory biochemistry including C-reactive protein (CRP), D-Dimer, lactate dehydrogenase (LDH), ferritin and complete blood count were evaluated, and treatment to be taken at home prescribed as per randomization. The SC medications as per the hospital guidelines included analgesics/anti-fevers and antibiotics. The quercetin complementary treatment consisted of an oral 500 mg Quercetin Phytosome® (developed by Indena S.p.A, Milan, Italy) tablets (Quevir®, manufactured by Pharmextracta S.p.A, Italy), 1^st^ week TDS, 2^nd^ week BDS as an add-on to the same SC. Each 500 mg Quercetin Phytosome® Quevir® tablet contained 200 mg pure quercetin formulated with sunflower lecithin. Patients were provided with a telephone number to contact the trial clinical team in case of an emergency or if they need any medical advice. A second in-person follow-up appointment was made for all the patients with the outpatient’s physician at day 7 of the treatment to test for the SARS-CoV-2 by RT-PCR and assess persistence of COVID-19-associated acute symptoms and evaluate biochemistry. If necessary, a 3^rd^ RT-PCR was also arranged for day 14^th^ and 21^st^ of the enrollment.

## Outcome

The study primary outcomes were to investigate the effect of quercetin supplementation on time needed to test negative for SARS-CoV-2 by RT-PCR analysis, and improvement in the COVID-19- associated acute symptoms, while secondary outcome include improvement in the laboratory biochemistry.

## Statistical analysis

For statistical evaluation between the two treatment groups, and within observation periods (Day 1 vs. Day 7), the Split-Plot Design, a mixed analysis of variance was used. In the presence of significant interaction, the Tukey’s Multiple HSD Comparison Test was applied. Statistical significance was achieved with *p* < 0.05. The variables analyzed with these procedures were CRP, LDH, Ferritin and D-Dimer. For SARS-CoV-2 RT-PCR analysis, Pearson’s Chi-Square statistic was used. To investigate if the patient’s age in the two groups could influence the results, the analysis of covariance (ANCOVA) was applied. To evaluate the significance of symptoms’ shift, the pseudo discrete-time Markov chain (DTMC) was applied. A contingency table was used with the change in symptoms’ frequency between Day 1 and Day 7. The DTMC analysis allowed us to define the subjects as healthy (patients who show one or more symptoms at baseline, but no symptoms at Day 7), improved (patients who show fewer symptoms at Day 7 than those at baseline), unchanged (patients whose symptoms were not changed by Day 7); worsened (patients with a higher number of symptoms at Day 7 than baseline). The results obtained from this methodology were compared, between groups, using Chi-Square Test (Pearson and Likelihood Ratio). Moreover, for a multivariate analysis of the results, all the variables were used simultaneously by Discriminant Analysis. The observed values of the two groups were represented as points (canonical variables) on a biplot graph. A 95% confidence level ellipse is plotted for each mean. If two groups differ significantly (*p* < 0.05), the confidence ellipses tend not to intersect.

## Results

Amongst the total 108 patients randomized, 100 patients consisting of 50 patients in the quercetin group and 50 patients in the control group, completed the study. Amongst the remaining 8 patients, 3 patients failed to attend the follow-up appointment and 5 patients discontinued the treatment on their own choice. Patients’ demographics and baseline clinical characteristics are summarized in Table 1. The mean age was 47.6 ± 15.7 years and includes almost half males and half females. The modal age group was between 30–40 years with 23% of the total cases (data not shown). At baseline, the two groups were overlapping for all parameters except age which on average was significantly higher in the control group. Patients on antihistamine drugs, statins, metformin, proton pomp inhibitors, acetyl salicylic acid, and antihypertensive drugs, were also not significantly different in the two groups (data not shown). All the patients in both groups had not received COVID-19 vaccination.

**TABLE 1:** Patient’s demographics and baseline clinical characteristics.

Characteristics	Control group (n = 50)	Quercetin group (n = 50)	*p*-value
Gender, male, female (n)	25, 25	23, 27	n.s
Age, mean ± SD (years)	54.1 ± 2.03	41.1 ± 2.03	<0.001
Comorbidities* (n)			
None/single	32	35	n.s
Two or more	18	15	n.s
COVID-19 symptoms (n)			
Fever^	43	41	n.s
Cough	29	28	n.s
Myalgia	28	29	n.s
Sore throat	24	23	n.s
Dyspnoea	12	9	n.s
Headache	1	3	n.s
Flu-like symptoms	5	6	n.s
Weakness	4	3	n.s

SD, standard deviation; n.s., not significant; *, hypertension; type 2 diabetes, heart disease, kidney disease, hypercholesterolemia, obesity, asthma/allergy; ^, no of cases with fever >39.5°C, average value for both groups was 38.7°C.

**FIGURE 3:**
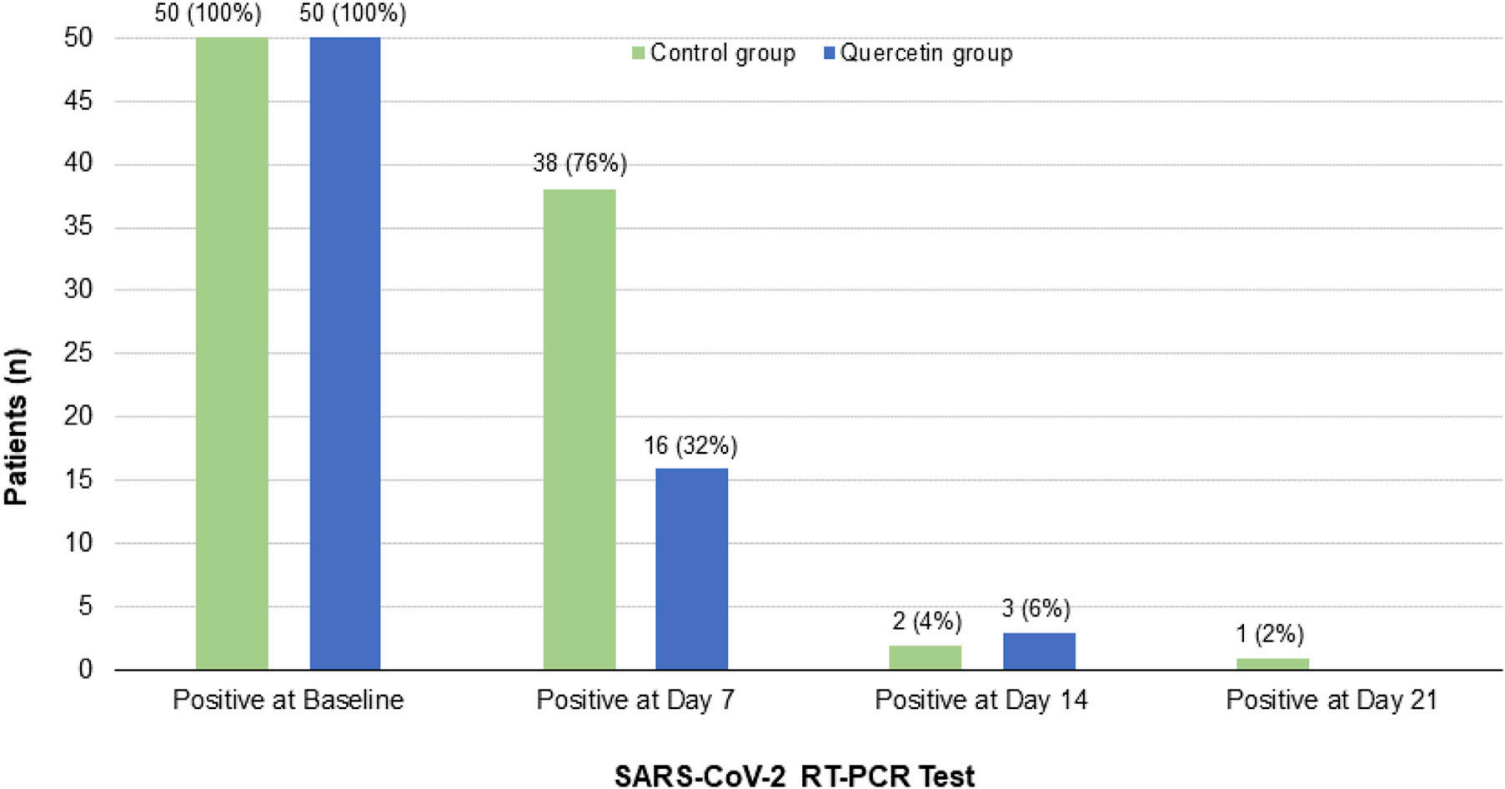
Patient’s follow-up RT-PCR COVID-19 test results in the two treatment groups.

In assessing the clinical recovery, by week one, the complementary treatment with quercetin significantly reduced the virus persistence by 68% vs. 24% in the control group (*p* = 0.0004) i.e, tested negative for SARS-CoV-2 (Figure 3). By week two, nearly all the subjects of both groups (98% in quercetin group, and 94% in the control group) tested negative for SARS-CoV-2. By week three, one patient was still SARS-CoV-2 positive in the control group, while there was no SARS-CoV-2 positive patient left in the quercetin group.

**FIGURE 4:**
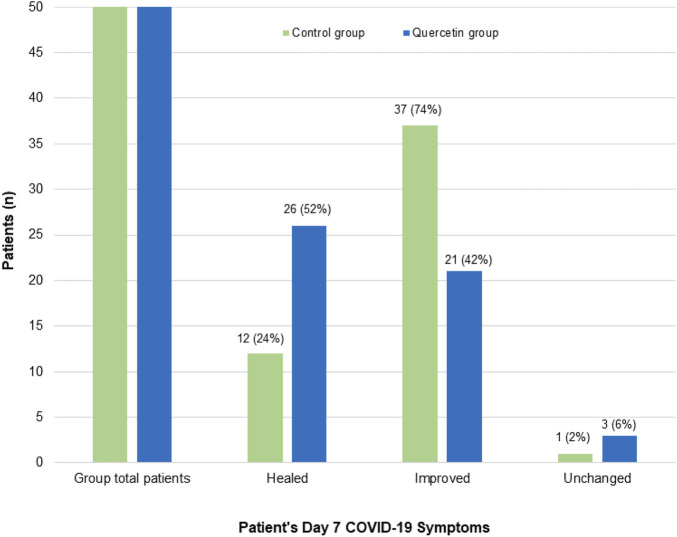
Patient’s Day 7 follow-up COVID-19-associated symptoms in the two treatment groups. Healed: Patients who manifested one or more COVID-19 associated symptoms at baseline, but no symptoms on Day 7; Improved: Patients who showed fewer symptoms on Day 7 than baseline; Unchanged: Patients who have no treatment effect on their COVID-19 symptoms.

In assessing the treatment effect on COVID-19-associated acute symptoms (Figure 4), significantly speedy clinical recovery was observed in the quercetin group, with 52% of patients had all their COVID-19-associated symptoms resolved by week one (vs. 24% in the control group) (*p* = 0.0051). By week two of treatment, most of the patients in both groups were free from COVID-19 symptoms (data not shown).

In evaluating the treatment effect on the patient’s serum levels of inflammatory biomarkers after one week of treatment, patients in the quercetin group showed a significant reduction in LDH levels (Table 2). Improvement in CRP, ferritin, and D-dimer levels were also observed but not significantly so. There was no change in the patient’s hemoglobin, platelets, white blood cells, neutrophils, and leucocytes counts at baseline and day 7 (data not shown). As the two groups showed a significant difference in age, a covariance analysis was performed to evaluate if this parameter could have affected the results. However, no such association was observed (data not shown).

**TABLE 2:** Patient’s baseline and day 7 inflammatory biomarkers levels in the two treatment groups.

Characteristic	Control group (*n* = 50)	Quercetin group (*n* = 50)	*p*-value
LDH (mean ± SD) U/L			
Baseline	359.28 ± 140.40	406.56 ± 183.92	0.0001
Day 7	316.84 ± 133.02	257.74 ± 110.73	
CRP (mean ± SD) mg/L			
Baseline	30.01 ± 27.40	25.93 ± 25.78	n.s
Day 7	17.36 ± 22.54	11.07 ± 13.65	
Ferritin (mean ± SD) ng/mL			
Baseline	639.17 ± 799.96	521.82 ± 249.91	n.s
Day 7	508.76 ± 594.72	306.48 ± 144.53	
D-Dimer (mean ± SD) ng/mL			
Baseline	266.13 ± 220.32	217.25 ± 69.67	n.s
Day 7	180.46 ± 102.40	186.14 ± 51.87	

LDH, lactate dehydrogenase; CRP, C-reactive protein. n.s., not significant.

One patient (sex: female; age: 69; affected by hypertension, type 2 diabetes, and chronic kidney disease) in the control group died (day 20 of enrolment) after being hospitalized and admitted in intensive care unit. Quercetin complementary treatment was well tolerated by all 50 patients with no apparent toxicity; and no peculiar side effects were reported by the patients.

**FIGURE 5:**
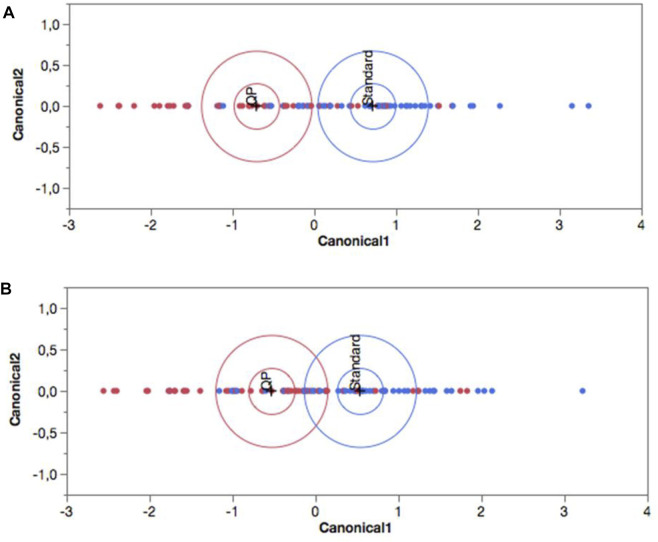
Canonical plot of the two treatment groups obtained by Discriminant Analysis at **(A)** baseline and **(B)** Day 7. Blue (standard of care alone), and red (with Quercetin Phytosome®) colours represents control and quercetin group respectively. The observations and the multivariate means of each group are represented as points on the biplot and expressed in terms of the first two canonical variables. The point corresponding to each multivariate mean is denoted by a plus (”+“) marker. A 95% confidence level ellipse is plotted for each mean. If two groups differ significantly, the confidence ellipses tend not to intersect. An ellipse denoting a 50% contour is plotted for each group. This depicts a region in the space of the first two canonical variables that contains approximately 50% of the observations, assuming normality.

To test the validity of our results, Discriminant Analysis were performed. The procedure divides patients into different groups according to their level of similarity measured by the values of simultaneously selected variables. As shown in Figure 5A, at baseline the two groups were overlapping without any clear differentiation. Therefore, according to the overall set of variables considered simultaneously, they were homogeneous. As shown in Figure 5B, by week one of the treatment, the two groups were no longer overlapping and showed a significant difference (*p* < 0.05; confidence level ellipse) due to the different response in the quercetin group to the variables considered as a whole.

## Discussion

This randomized clinical trial investigated the possible adjuvant effect of an oral quercetin supplementation in mild to moderately symptomatic COVID-19 outpatients. The results revealed that patients who received quercetin in addition to SC, expeditiously cleared the virus (tested negative for SARS-CoV-2) (*p* = 0.0004) and had a speedy resolution of their COVID-19-associated acute symptoms as compared to the patients who received the SC alone (*p* = 0.0051). Moreover, patients in the quercetin group also showed statistically significant improvement in the serum levels of inflammatory biomarker LDH, *p* = 0.0001. In this study no treatment-emergent effect or serious adverse events were reported which further confirm the safety of quercetin in patients with COVID-19. The speedy clearance of the SARS-CoV-2 in patients in the quercetin group is possibly due to the inhibition of the SARS-CoV-2 3C-like protease (3CL_Pro_), also called main protease (M_pro_) by quercetin (Chen et al., 2006;; Kandeil et al., 2021; Rizzuti et al., 2021; Bahun et al., 2022). 3CL_Pro_ is a crucial enzyme involved in the replication of SARS-CoV-2. Quercetin can also exert its antiviral effect by acting either directly on the viral particles i. e; blocking the viral cell entry (Yi et al., 2004; Wu et al., 2015) or at difference stages of the replecative cycle by interacting with the viral proteins or by interferring in the cellular processes of pathways essential for the viral replication (Ono and Nakane, 1990; Chiang et al., 2003). Human angiotensin-converting enzyme 2 (ACE2) has been identified as a receptor of S-protein of SARS-CoV-2 to infiltrate host cells (Lan et al., 2020; Yan et al., 2020); inhibiting the interaction between S protein and the host ACE2 is believed to inhibit SARS-CoV-2 infection. Quercetin has been reported to be an inhibitor of ACE2 (Liu et al., 2020).

The present study also revealed significant improvement in the patients serum LDH levels, supporting the possible anti-inflammatory therapeutic effect of quercetin in COVID-19. Serum LDH level is an important severity marker in patients with COVID-19 (Szarpak et al., 2021). In a metanalysis study by Henry et al. (2020) elevated LDH levels were associated with a ∼6-fold increase in odds of developing severe disease and a ∼16-fold increase in odds of mortality in patients with COVID-19. Quercetin exhibits diverse anti-inflammatory mechanisms including the inhibition of lipid-peroxidation, lipoxygenase and phospholipase A2, the nucleotide-binding oligomerization domain leucine rich repeat and pyrine domain containing protein 3 (NLRP3) inflmmasome-mediated IL-1ß production (Tőzsér and Benkő, 2016), and pro-inflammatory cytokines such as IL-1ß, IL-6, INF-γ, and TNF-α (Colunga Biancatelli et al., 2020). Quercetin in combination with dasatinib has been shown to selectively eliminate SARS-CoV-2 virus-induced senescent cells, mitigate COVID-19 reminiscent lung disease, and reduce inflammation in SARS-CoV-2 infected hamsters and mice (Lee et al., 2021). Systemic inflammation, which is predominant in the lung cells is a hallmark of severe COVID-19; and anti-inflammatory therapies including corticosteroids, interleukin-6 (IL-6) receptor blockers (tocilizumab) and Jinas-kinase inhibitors (bariticinib) have been shown to help in reducing mortality in hospitalized severely-ill COVID-19 patients (Kalil et al., 2020; RECOVERY Collaborative Group, 2021a; RECOVERY Collaborative Group, 2021b). These anti-inflammatory therapies however have shown no treatment benefits in the early stage of COVID-19. The clinical course of SARS-CoV-2 infection is quite unpredictable particularly in vulnerable patients; and a combination of suitable antiviral and anti-inflammatory therapies in the early-stage is believed to be the key in preventing the progression to severe illness. We can therefore, suggest that the complementary therapy of quercetin has the potential to interfere with the early stages of the SARS-CoV-2 infection (replication), and simultaneously helping in the modulation of the host’s hyperinflammatory response. Together with these effects quercetin supplementation can help in the speedy recovery of patients from COVID-19. Moreover, unlike the conventional anti-inflmammatory agents, quercetin does not suffer from the intrinsic drawbcak of immuno-supression.

The results of quercetin complementary therapy revealed in this study are also in line with those reported by other randomized clinical trials in hospitalised COVID-19 patients. In study by Shohan et al. (2022) a daily 1000 mg quercetin supplementation with antivirals (remdesivir or favipiravir) for 7 days was significantly associated with partial earlier hospital discharge, reduction in the serum levels of LDH, alkaline phosphatase (ALP), and q-CRP, as well as significant increase in the patients hemoglobin level and respiratory rate as compared to the antiviral treatment alone. In study by Onal et al. (2021) a daily 1000 mg of quercetin (in combination with 1000 mg vitamin C and 100 mg bromelain) resulted in significant reduction in CRP, ferritin levels, and increase in platelet and lymphocyte counts. In study by Zupanets et al. (2021) on COVID-19 patients associated with pneumonia, intravenous administration of quercetin/polyvinylirolidone during the first 10 days followed by oral administration of quercetin/pectin over the next 10 days, significantly improved the patients oxygen saturation level, cough, as well as helped to stabilize the level of D-dimer. Moreover, quecetin has also been assessed as possible proplylactic for COVID-19. In study by Rondanelli et al. (2022) a daily 500 mg quercetin supplementaion provied 14% additional protection to volunteers against SARS-CoV-2 infection as compared to the placebo group.

Although several COVID-19 vaccines have been developed, and the rate of SARS-CoV-2 infection and community spread, hospitalization and mortality have significantly reduced, but the pandemic continues and affecting the lives of everyone. There is currently a fresh wave of COVID-19 in many developing countries with still a higher rate of hospitalization and mortality as compared to the developed nations. To protect the world’s population from COVID-19 infection, would require entire world’s population to be COVID-19 vaccinated. However, such efforts could take many years and have many associated challenges including cost, distribution under cold-chain, anti-vaccine campaign, and emergence of new SARS-CoV-2 variants due to mutations etc. Therefore, there is an urgent need of safe, affordable, and worldwide available medications for COVID-19 to treat the disease in the early-stage and prevent community transmission. Although scientific efforts to date have been more focused on drug identification (repurposing) for the treatment of hospitalized COVID-19 patients with the disease already in the advance stage; identification of treatment for early-stage and/or mild disease is critical to curb the COVID-19 transmission. Treating people early in the course of infection with SARS-CoV-2, would speed their recovery, reduce the likelihood that they develop severe outcomes and reduce demand on the healthcare system. In this context quercetin is a safe, cheap, and worldwide available agent; and in combination with SC can help and contribute in the efforts to control the ongoing pandemic.

Our study also has limitations. Small sample size, and not being in a double-blind and placebo-controlled conditions are some of the drawbacks of our study. Nevertheless, we are in a pandemic and there is an urgent need of safe, cheap, and readily available medications for COVID-19, particularly in the developing countries. We have carried out a pragmatic clinical study to investigate the possible complementary treatment benefits of quercetin in patients with early-stage COVID-19. The results revealed in this study can be easily translated to help in this pandemic.

In conclusions, the results revealed in this study suggest possible therapeutic role of quercetin supplementation in the early-stage mild to moderately symptomatic COVID-19 outpatients and may help in the speedy clearance of the SARS-CoV-2 infection, early resolution of the acute symptoms and modulation/control of the host’s hyperinflammatory response. The present study also supports the safety of quercetin supplementation in patients with COVID-19 which has an unpredictable and complex course and hence may be used as an adjuvant alongside routine care in the management of mild to moderate symptoms of COVID-19.

## Data Availability

The original contributions presented in this study are included in this article, further inquiries can be directed to the corresponding authors.
